# Miransertib (ARQ 092), an orally-available, selective Akt inhibitor is effective against *Leishmania*

**DOI:** 10.1371/journal.pone.0206920

**Published:** 2018-11-06

**Authors:** Devki Nandan, Naixin Zhang, Yi Yu, Brian Schwartz, Stella Chen, Peter E. Kima, Neil E. Reiner

**Affiliations:** 1 Department of Medicine, Division of Infectious Diseases, University of British Columbia, Vancouver, BC, Canada; 2 Department of Microbiology and Cell Science, University of Florida, Gainesville, Florida, United States of America; 3 ArQule, Inc, Burlington, Massachusetts, United States of America; 4 Department of Microbiology and Immunology, University of British Columbia, Vancouver, BC, Canada; Taibah University, SAUDI ARABIA

## Abstract

Leishmaniasis is amongst the most important neglected diseases, afflicting more than 12 million people in 88 countries. There is an urgent need for safe orally bioavailable and cost-effective drugs for the treatment of leishmaniasis. It has recently been shown that *Leishmania* activates host macrophage serine/threonine kinase Akt, to promote survival of both parasites and infected cells. Here, we sought to evaluate a compound, Miransertib (ARQ 092), an orally bioavailable and selective allosteric Akt inhibitor currently in clinical trials for patients with PI3K/Akt-driven tumors or Proteus syndrome. Miransertib was tested against *Leishmania donovani* and *Leishmania amazonensis*, causative agents of visceral and cutaneous leishmaniasis, respectively. Cultured promastigotes were susceptible to Miransertib. In addition, Miransertib was markedly effective against intracellular amastigotes of *L*. *donovani* or *L*. *amazonensis*-infected macrophages. Miransertib also enhanced mTOR dependent autophagy in *Leishmania*-infected macrophages, which may represent one mechanism of Miransertib-mediated killing of intracellular *Leishmania*. Whereas parasite clearance in the spleen of mice infected with *L*. *donovani* and treated with Miransertib was comparable to that when treated with miltefosine, Miransertib caused a greater reduction in the parasite load in the liver. In the cutaneous leishmaniasis infection model, lesions were reduced by 40% as compared to mock treated mice. Together, these results provide direct evidence to support the conclusion that Miransertib is an excellent lead compound for the development of a new oral drug therapy for visceral and cutaneous leishmaniasis.

## Introduction

Leishmaniasis is caused by protozoan parasites of the genus *Leishmania* that are transmitted by the bite of infected female sand flies. Currently, over 350 million people are at risk for leishmaniasis [1]. There is no effective vaccine against leishmaniasis, and drugs are the only available tools to treat and control leishmaniasis. Only a limited number of drugs (such as pentavalent antimony (SbV) compounds, amphotericin B, pentamidine and miltefosine) are available to treat leishmaniasis. The development of parasite resistance to drugs currently in use is a concern [2]. In addition, most of the drugs currently in use have a narrow therapeutic index, cause severe side-effects, and require long-term treatments [3]. Thus, there is an urgent need to develop new therapeutics.

In mammalian hosts, *Leishmania* are intracellular pathogens that primarily infect macrophages and also other phagocytic cells including dendritic cells and neutrophils. *Leishmania* has a profound effect on the cell biology of its host cell, including the suppression of signal transduction that can lead to cell activation and the suppression of pro-inflammatory responses [4]. Recently, we [5] and others [6–8] have shown that *Leishmania* activates Akt in infected cells. Akt phosphorylates Ser/Thr residues on a variety of downstream targets including Glycogen Synthase Kinase -3 beta (GSK-3β), Forkhead Box O (FOXO1) and Bcl-2-associated death promotor (BAD), which regulate cellular processes such as cell growth, survival, and metabolism [9,10]. A few reports have shown that Akt is activated by *Leishmania* infection and that knock down of Akt limits survival of *Leishmania* parasites in infected cells [6,11–13], which suggests that Akt plays an essential role in *Leishmania* pathogenesis. Human Akt isoforms share around 98% sequence homology with mouse orthologs predicting relevance of murine models of leishmaniasis to human application. In light of the fact that several Akt inhibitors are being developed as anti-cancer drugs [14,15] we elected to evaluate the recently described compound, (3-(3-(4-(1-Aminocyclobutyl)phenyl)-5-phenyl-3Himidazo[4,5-b]pyridin-2-yl)pyridin-2-amine) named Miransertib (ARQ 092). Miransertib has been shown to be a highly-selective Akt inhibitor which can be administered orally [16]. It inhibits the activity of all three Akt isoforms [17]. It has been proposed that Miransertib blocks membrane translocation of inactive Akt and promotes dephosphorylation of the membrane-associated active form, thereby attenuating Akt activity [16].

In the present study, we demonstrate that Miransertib markedly controlled infections of *L*. *donovani* and *L*. *amazonensis*. This effect was selective as Miransertib was not toxic to macrophages as revealed by MTS assay, which measures the metabolic status of the cell. The excellent selectivity and anti-leishmanial activity of Miransertib *in vitro* prompted the evaluation of its anti-leishmanial activity *in vivo* in BALB/c mice infected with either *L*. *donovani* or *L*. *amazonensis*. *In vivo* studies revealed that oral administration of Miransertib to *L*. *donovani* infected mice effectively reduced the parasite burden in livers and spleens. In experimental infections with *L*. *amazonensis*, it significantly reduced cutaneous lesions. Taken together, these results strongly suggest that Miransertib warrants further study and should be included in clinical trials of leishmaniasis.

## Material and methods

### Test compound, reagents and antibodies

Miransertib (ARQ 092) was supplied by ArQule Inc. (Burlington, MA, USA) and Miltefosine was obtained from Cayman Chemicals (Ann Arbor, Michigan). Antibodies for LC3-II, Akt, and phospho-Akt (S473) were obtained from Cell Signalling Technologies (Danvers, MA). Promega CellTiter 96 AQueous One Solution Cell Proliferation Assay (MTS) was from Fisher scientific.

### Parasite culture

Sudan strain S2 of *L*. *donovani* promastigotes (kindly provided by K.-P. Chang, Rockefeller University, New York) and also, *L*. *donovani* (MHOM/S.D./62/1S‐CL2D) obtained from Nakhasi's lab were incubated and cultured at 26°C in M199 media as described [18].

The *L*. *amazonensis* strain RAT/BA/74/LV78 (LV78) was cultivated in Schneider’s Drosophila Medium (BioWhittaker) as described [19].

### Mice

Female BALB/c mice were obtained from Charles River Laboratories, Wilmington, MA. The animals were maintained under specific pathogen-free conditions.

### THP-1 cell culture, differentiation and *Leishmania* infection

THP-1 cells (human monocytic cell line), obtained from ATCC (TIB-202TM), were incubated and cultured as previously described [18]. For differentiation, THP-1 cells were treated with 10 ng/mL PMA for 16–18 hours as described [18]. For infections, day 5 stationary promastigotes were used at an MOI of 20:1.

### Mouse macrophage culture and infections

RAW264.7 (murine macrophage cell line) cells were obtained from ATCC and maintained in DMEM medium as described [6]. For *L*. *amazonensis*, infections were initiated with stationary phase promastigotes at 1:5 or 1:20 ratio for 24 hours. After 24 hr, infections were treated with concentrations of ARQ092 in the range of 0 to 5 μM or miltefosine in the range of 0 to 10 μM and incubated for an additional 24 hours. Coverslips were then washed and treated with the varying concentrations of Miransertib or miltefosine. Some coverslips were incubated in DMSO as vehicle control. Coverslips were fixed in methanol and infected cells were stained with Wright-Giemsa stain as described [20].

### Western blot

Cells were washed with HBSS and lysed in ice-cold cell lysis buffer (20mM Tris-HCl pH 7.2, 1% Triton X-100, 1 mM EDTA, 0.15 M NaCl), containing a cocktail of protease and phosphatase inhibitors. Whole cell lysates were separated by SDS-PAGE and transferred to appropriate transfer membranes (Bio-Rad) using a semi-dry blotter. For LC3-II, whole cell lysates were subjected to Tris-tricine, 15% SDS-PAGE and transferred onto PVDF membrane. For pAkt and Akt, whole cell lysates were subjected to Tris-glycine, 10% SDS-PAGE and transferred to nitrocellulose membrane. Transferred proteins on membranes were probed with appropriate antibodies, according to the manufacturer’s instructions. Briefly, for anti-LC3-II, 5% non-fat milk +3% BSA were used to block non-specific antibody binding. For anti-pAkt and Akt, 5% BSA was used as a blocking agent. Membranes were incubated with the following primary antibodies: anti-LC3-II (Cell Signaling, 1:1000 dilution in blocking solution), pAkt (Cell Signaling, 1:1000 in blocking solution) and Akt (Cell Signaling, 1:3000 in blocking solution) with gentle agitation over night at 4^°^C. Following primary antibody incubation and washes, secondary antibody used was anti-rabbit IgG-HRP (Sigma). Protein bands were observed on Blue X-ray Film (Carestream) using ECL Select Western Blotting Detection Reagent from GE Healthcare (RPN2235) for enhanced chemiluminescence. Densitometric analysis was performed in ImageJ.

### MTS assay

This colorimetric assay was performed for sensitive quantification of viable cells in proliferation according to manufacturer’s instructions. Briefly, THP-1 cells were PMA differentiated at a density of 3 × 10^5^ cells/mL in 500 μL of complete RPMI in 24-well plates at 37°C, and then treated with various concentrations of Miransertib for 24 hours. Next, One Solution reagent was added to the dTHP-1 cells to a final concentration of 10% v/v, and the plates were then incubated at 37°C for an additional 4 hours. The absorbance at 490 nm was recorded using an ELISA plate reader. All experiments were performed in duplicate and individually repeated three times.

### Parasite rescue and transformation assay

For this assay, infected cells were extensively washed with HBSS to remove non-internalized parasites. Controlled lysing of infected cells was performed using 0.01% SDS as described previously [18,21]. Quantification of the infection was performed through transformation of live, rescued *Leishmania* amastigotes to log phase promastigotes in growth media (M199) by incubating the plates in 26°C for 48 hours. The evaluation of their growth was performed by manual counting of transformed promastigotes.

### *In vivo* Miransertib sensitivity in visceral leishmaniasis model

Eight-week-old groups of female BALB/c mice (4 per group) were inoculated intravenously (tail vein) with 1 x 10^7^ stationary phase *L*. *donovani*. Starting from day 7 post-infection, groups of mice were treated with either drug vehicle only (orally), with miltefosine (20 mg/kg orally), or with Miransertib (50 mg and 75 mg/kg orally). Miltefosine and Miransertib were administered once daily for 5 days per week for 4 weeks. At the end of the study, all animals were humanely euthanized and parasite burdens were determined by counting the number of rescued viable amastigotes from infected liver and spleen.

### *In vivo* Miransertib sensitivity in cutaneous leishmaniasis model

Six-week-old groups of female BALB/c mice (5 per group) were inoculated with approximately 1 x 10^7^ stationary phase *L*. *amazonensis* in the back of the left hind foot. From day 1 post-infection, groups of mice were administered with the following compounds orally: saline, miltefosine (8 or 16 mg/kg), Miransertib (50 and 100 mg/kg) for 5 days. Measurements of infected and contralateral foot were made with a Pocket dial thickness gage (C3, Chicago Brand).

### Ethics statement

After arrival, mice had 1 week of pre-adaptation period. The mice were housed four per cage in level 2 containment and maintained at a constant temperature. After that 1 week of acclimation, the mice were randomly divided into various groups. Prior to inoculation with parasites, mice were lightly anesthetized. The animals had free access to water and food. They were given mouse igloos or tubes for shelter and received crinkly paper for nesting material. Body weight was measured daily during the experimental course. Animals were monitored daily to assess health and well-being. The main distress criteria were loss of weight, dehydration, diarrhea, hyperactivity and ruffled fur. No loss in animal body weight and no sign of morbidity were detected during the period of drug treatment. Animals were euthanized, following institutional guidelines, 24 hours after the last drug administration. The animals were anesthetized with isoflurane prior to CO_2_ euthanasia. The infected organs were quickly removed, weighed and subsequently processed for parasite load evaluation.

### Ethical approval

Prior approval for animal experiments was obtained from the Animal Care Committee of University of British Columbia (approval# A14-00218) and also from IACUC at the University of Florida.

### Evaluation of autophagy in macrophages from *L*. *donovani* infected spleens

For the evaluation of autophagy, macrophages from a portion of mouse spleen were prepared as follows. Spleens from control and infected mice were separately pressed through 40 μm pore size cell strainers. The cell suspensions were centrifuged at 300 x g at room temperature for 10 minutes. Equal numbers of spleen cells from control and infected mice were transferred to 6-well cell culture dishes containing IDMEM complete growth media. Cells were incubated at 37°C, 5% CO_2_ for 2 hours. Non adherent cells were removed by extensive washings. Macrophages remaining attached were lysed and assessed for LC3-II levels by Western blotting.

### Statistical analysis

The data of three independent experiments were determined using paired, t-test on GraphPad Prism 6.0 Software. The values were considered statistically significant at *p < 0.05, **p < 0.01, ***: p<0.001.

## Results

### Inhibition of *Leishmania* growth by Miransertib *in vitro*

**Fig 1:**
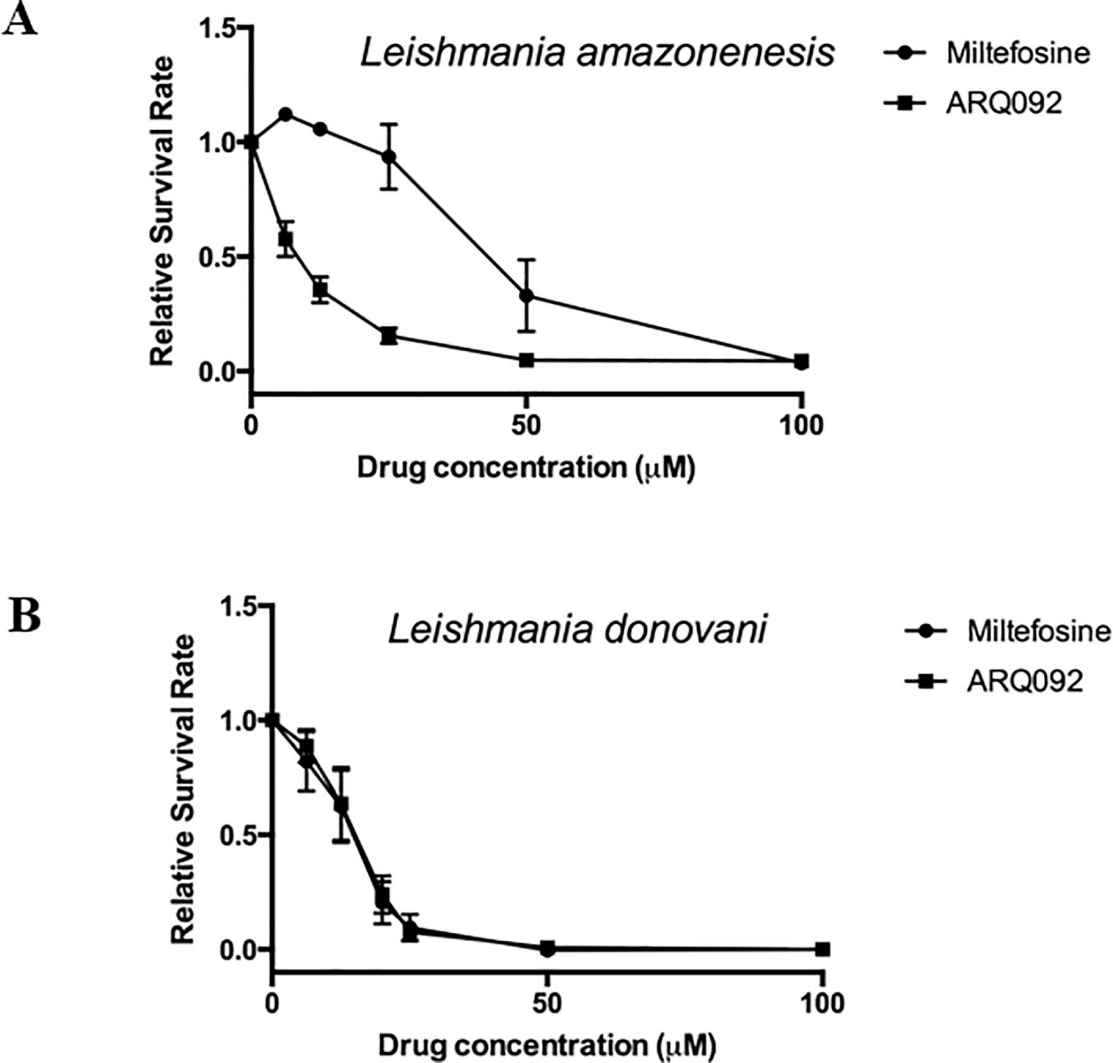
The efficacy of Miransertib (ARQ092) as compared to miltefosine on promastigote forms of *Leishmania amazonensis* (**A**) and *Leishmania donovani* (**B**) was measured in MTT assays after 24 h treatment of each drug at increasing concentrations. The proportion of surviving parasites was analyzed by GraphPad prism and the EC_50_ was calculated from those plots. The data was compiled from at least 3 independent experiments.

To determine the inhibitory effect of Miransertib on *Leishmania* growth, we studied its effect on promastigotes of *L*. *amazonensis* and *L*. *donovani*. First, promastigotes of *L*. *amazonensis* were treated with various concentrations of Miransertib for 24 hours. The number of viable parasites was determined by MTT assays. Fig 1 shows that increasing concentrations of Miransertib results in death of *Leishmania* parasites. The EC_50_ of Miransertib on *L*. *amazonensis* was estimated at 7.87 ± 0.93 μM (Fig 1A). For comparison, the EC_50_ of miltefosine on *L*. *amazonensis* was estimated at 40.83 ± 5.03 μM. The efficacy of Miransertib on *L*. *donovani* parasites was also determined to be 12.75 ± 1.14μM which was similar to the EC_50_ of miltefosine on *L*. *donovani*, which was 10.54 ± 3.51 μM (Fig 1B). These results show that Miransertib is as effective as miltefosine in killing *L*. *donovani* promastigotes, but it is slightly more effective on *L*. *amazonensis* promastigotes as compared to miltefosine.

### Miransertib reduces survival of intracellular amastigotes

**Fig 2:**
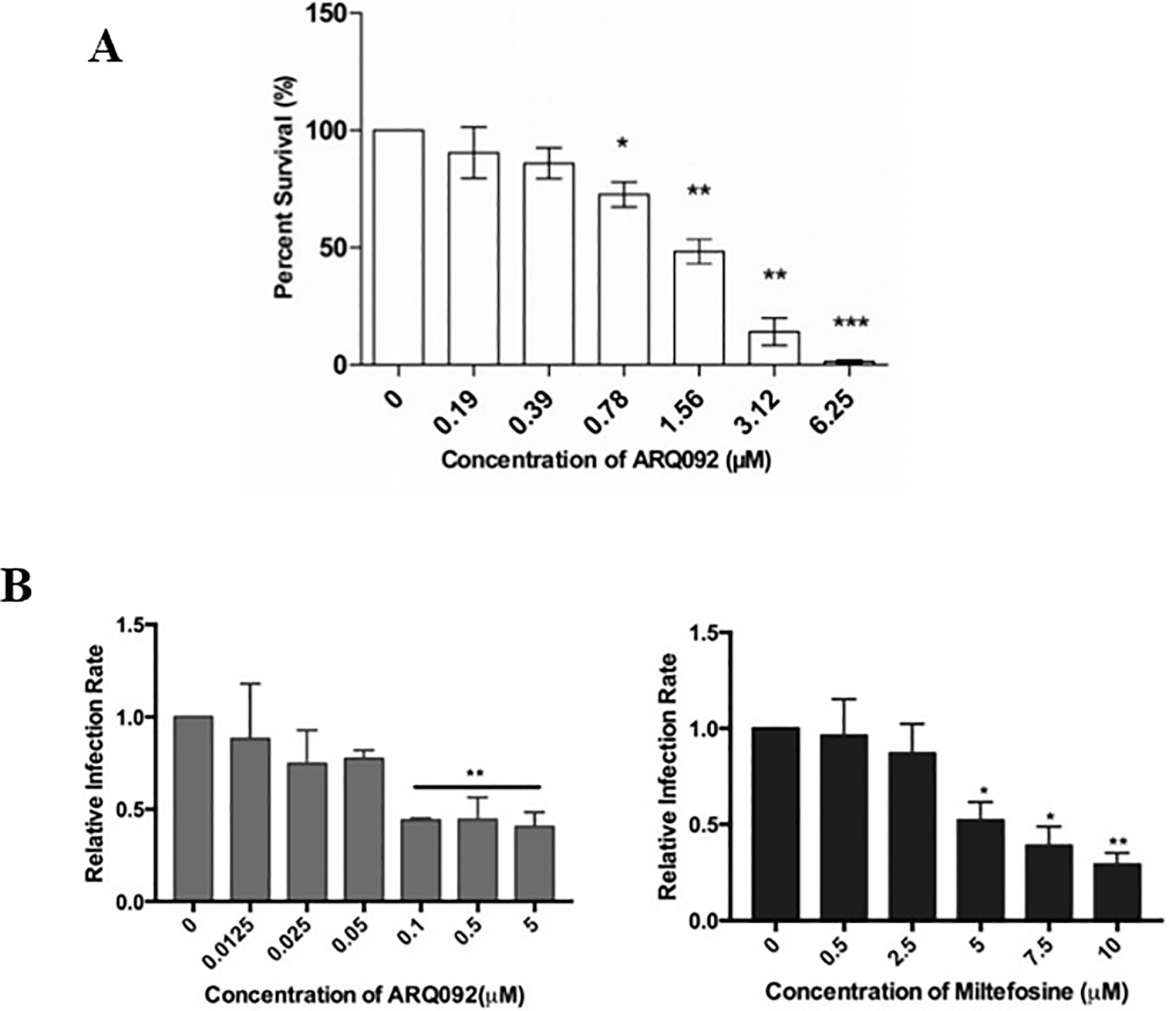
Determination of anti-leishmanial potential of Miransertib (ARQ092) on *Leishmania* in infected macrophages. **(A)** dTHP-1 cells (human macrophages) were infected with *L*. *donovani* promastigotes for 24 hours and then treated with indicated concentrations of Miransertib for an additional 24 hours. At the end of the experiment, infected cells with and without Miransertib treatment were washed, and internalized parasites were released by lysing the cells using a mild concentration of SDS, followed by transfer to transformation medium as described in “Materials and Methods”. Transformed motile promastigotes were counted. The data was compiled from three independent experiments done in duplicates. **B**) RAW264.7 macrophages were infected with *L*. *amazonensis* promastigotes. After 24 hours, infections cells were treated with indicated concentrations of ARQ092 or miltefosine and incubated for an additional 24 hrs. Cells on coverslips were Giemsa-stained and enumerated. The proportion of infected cells was compiled and plotted in GraphPad. Data were compiled from at least 3 experiments.

Before proceeding to assess the effect of Miransertib on intracellular parasites, we tested the inhibitory effect of Miransertib on Akt phosphorylation in differentiated THP-1 cells (dTHP-1). Cells were treated with either vehicle or the indicated concentrations of Miransertib for a period of 4 hours, followed by stimulation with 100 nM LPS, an activator of Akt [22], for 45 minutes. At the end of the experiment, whole cell lysates were analyzed for the level of phosphorylated Akt by immunoblotting using phospho-Akt antibody. As shown in **S1 Fig**, pre-treatment of dTHP-1 cells with Miransertib at various concentrations completely abolished the phosphorylation of Akt induced by LPS. This shows that Miransertib functions as a potent Akt inhibitor. We also examined whether the concentrations of Miransertib being used were sufficient to affect functional pathways regulated by Akt. We elected to examine autophagy, as Akt activation is known to inhibit autophagy and has been shown to regulate host macrophage autophagy in response to *Leishmania* infection [18]. dTHP-1 cells were treated with various concentrations of Miransertib for 4 hours, and whole cell lysates were analyzed for the induction of microtubule-associated protein 1 light chain 3b (LC3-II)/ATG8 which associates with autophagosomes. LC3-II has been used extensively as an indicator of autophagy in a wide variety of cells and tissues [23]. As shown in **S2 Fig**, Miransertib markedly enhanced the basal level of autophagy in dTHP-1 cells in a concentration dependent manner. To rule out the possibility that the indicated concentration of Miransertib used was cytotoxic to mammalian cells, the cytotoxic effect of Miransertib on dTHP-1 cells was also examined in MTS assays. The results shown in **S3 Fig** demonstrate that Miransertib was not toxic to mammalian cells at biologically active concentrations up to 12.5 μM. Comparable results were obtained with RAW264.7 macrophages on which no toxicity was observed below 10 μM. Thus, Miransertib concentrations in the non-toxic range were selected to evaluate its anti-amastigote efficacy in dTHP-1 cells infected with *L*. *donovani* and in RAW264.7 cells infected with *L*. *amazonensis*. Infections were initiated with promastigote forms, which transform to amastigotes after 16 to 19 hours [24]. After exposure to Miransertib for 24 hours, infections were either Giemsa-stained and scored microscopically or a parasite rescue and transformation assay was performed to determine the efficacy of Miransertib. The results presented in Fig 2A show clear anti-*Leishmania* activity of Miransertib on intracellular amastigotes of *L*. *donovani* in THP-1 cells. Killing of intracellular parasites occurs in a concentration dependent manner. In infections by *L*. *amazonensis* parasites in the RAW264.7 cells, the efficacy of Miransertib was evaluated in parallel experiments with miltefosine. Fig 2B shows that both Miransertib and miltefosine are effective in controlling *L*. *amazonensis* infection of the RAW264.7cells. The EC_50_ of Miransertib was 0.08 ± 0.07 μM as compared to miltefosine that was 4.27 ± 1.33 μM (miltefosine is not toxic to RAW264.7 macrophages at the concentration tested in this study, (13). Taken together, the data show that Miransertib has potent anti-leishmanial activity, in the low micromolar range, against intra-macrophage amastigotes forms of *L*. *donovani* and *L*. *amazonensis*.

### *In vivo* efficacy of Miransertib in a murine model of visceral leishmaniasis

The *in vivo* efficacy of Miransertib was assessed in a BALB/c model of visceral leishmaniasis. Prior to assessing *in vivo* efficacy of Miransertib in mice infected with *L*. *donovani*, the oral treatment regimens at 50 and 75 mg/kg body weight were tested on naive BALB/c mice (four mice in each group) for three weeks (five consecutive days per week) for tolerability. Neither loss of weight or other indicators of toxicity (as described in Ethics statement in “Materials and Methods” section) was observed in all Miransertib treated mice compared to untreated and vehicle treated mice.

**Fig 3:**
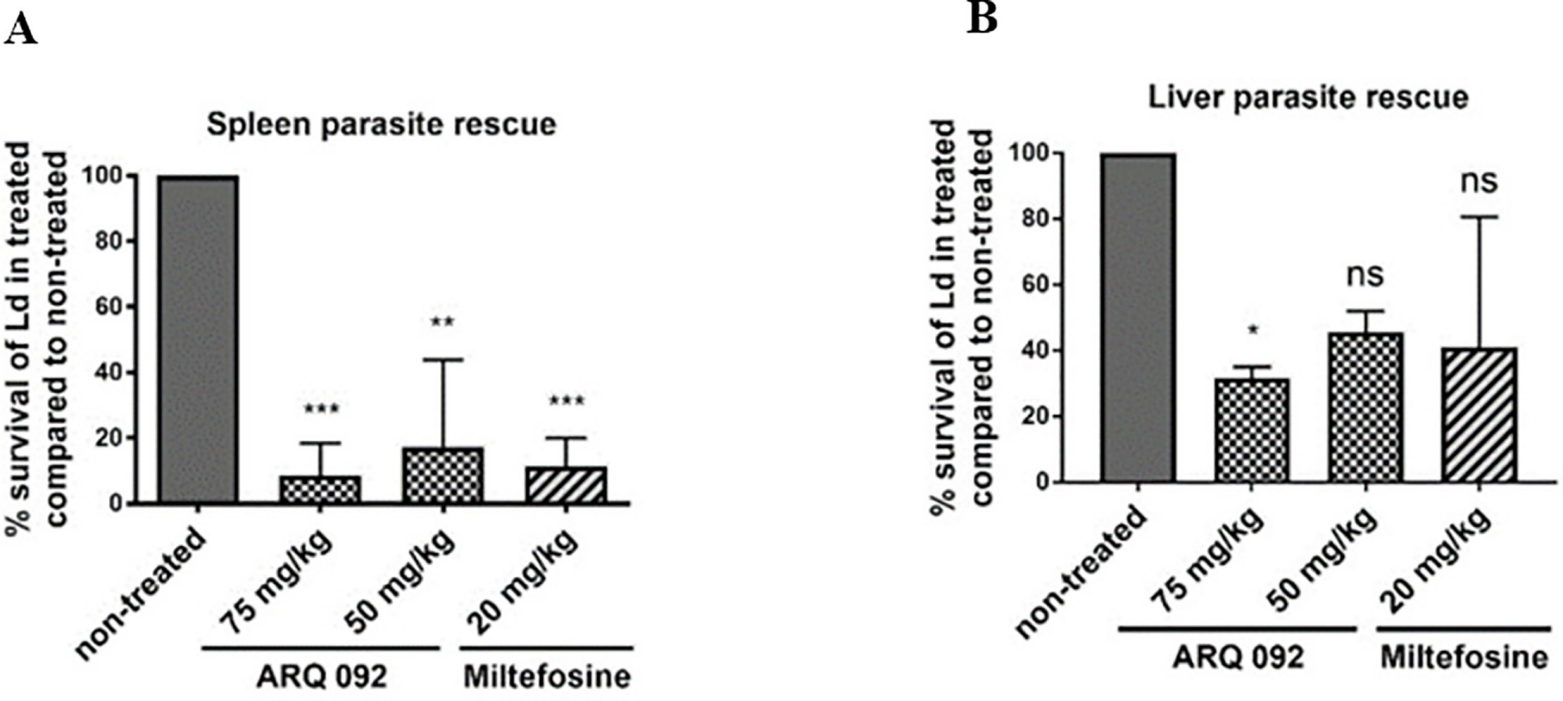
Effect of Miransertib on the parasitic burden in *L*. *donovani*-infected BALB/c mice. Female BALB/c mice (n = 4) were infected intravenously with approximately 10^7^ stationary phase *L*. *donovani* promastigotes on day -7. On day 0, treatment was begun with Miransertib (50 or 75 mg/kg) orally or 20 mg/kg of miltefosine orally. The control group received only vehicle orally. Drugs or vehicle were given five days a week for 4 weeks. The parasite survival in spleens (**A**) and livers (**B**) were determined at the end of the experiment as described in “Materials and Methods”. Significance of differences between non-treated and treated was tested by student t-test *p<0.05; **p<0.01 and***p<0.001.

For *in vivo* efficacy of Miransertib, groups of *L*. *donovani* infected BALB/c mice (seven days post infection with stationary phase promastigotes) were dosed daily, for five consecutive days per week with an oral formulation of Miransertib (50 and 75 mg/kg). On day 28 post-infection, the parasite burdens in the livers and spleens of infected mice were quantified. The only available FDA approved oral anti-leishmanial drug miltefosine (20 mg/kg, once daily, 5 days per week) was included as a positive control. Both Miransertib and miltefosine were well tolerated at both doses throughout the study, with no mice displaying any overt signs of toxicity. The results show that Miransertib effectively suppressed parasite burdens by 80–90% (Fig 3A and 3B). In fact, at 50 and 75 mg/kg, Miransertib effectively cured 50% of the study mice (2/4 mice in both treatment groups), with no detectable parasites in the livers and spleens. 50 and 75 mg/kg doses of Miransertib compared favorably with miltefosine in the spleen (Fig 3A), whereas it was more active than miltefosine in the liver (Fig 3B). These results show that Miransertib has potent anti-leishmanial activity *in vivo* against *L*. *donovani*.

### *In vivo* efficacy of Miransertib in a murine model of cutaneous Leishmaniasis

**Fig 4:**
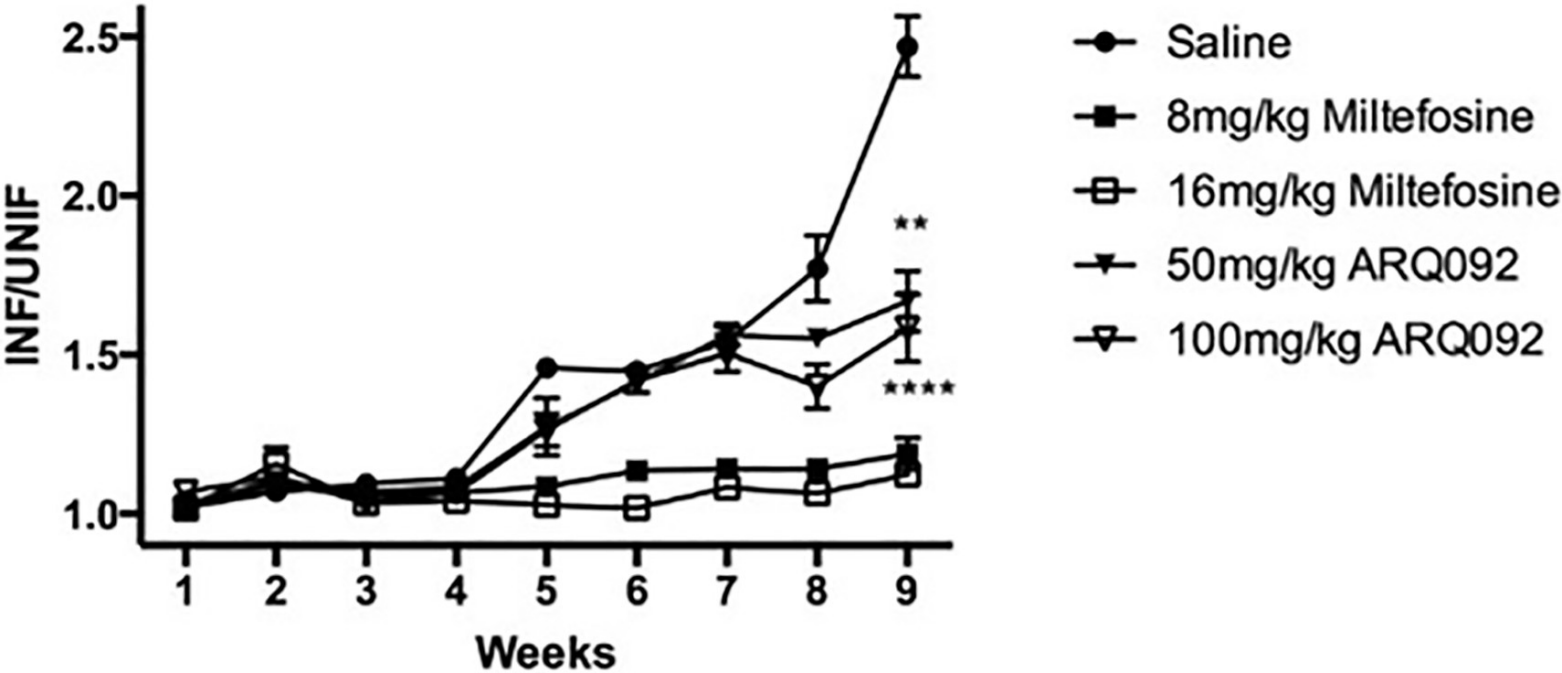
Miransertib partially controls *L*. *amazonensis* infection. Groups of 5 BALB/c mice were administered saline or the drug concentrations indicated orally for 5 days a week. Kinetics of infection was determined by weekly measurement of lesions in infected foot compared to the contralateral foot. Each point is the average ± SE **: p<0.005 ***: p<0.0005.

To evaluate the *in vivo* efficacy of Miransertib against *L*. *amazonensis*, infected BALB/c mice were orally administered with saline or either 50 mg/kg or 100 mg/kg of Miransertib daily for 5 days a week. Two other groups of mice were orally administered 8 or 16 mg/kg of miltefosine. Foot measurements were obtained every week for 9 weeks at which time mice were sacrificed. At the concentrations of drugs administered, no mice showed any signs of toxicity. The plots in Fig 4 show that the cutaneous lesions were reduced significantly after treatment with 50 or 100 mg/kg of Miransertib by 32 and 40%, respectively as compared to the saline group. Miltefosine treatment resulted in even greater reductions in lesions as compared to both the mice treated with saline and with Miransertib.

### Miransertib potentiates autophagy in *Leishmania* infected macrophages

**Fig 5:**
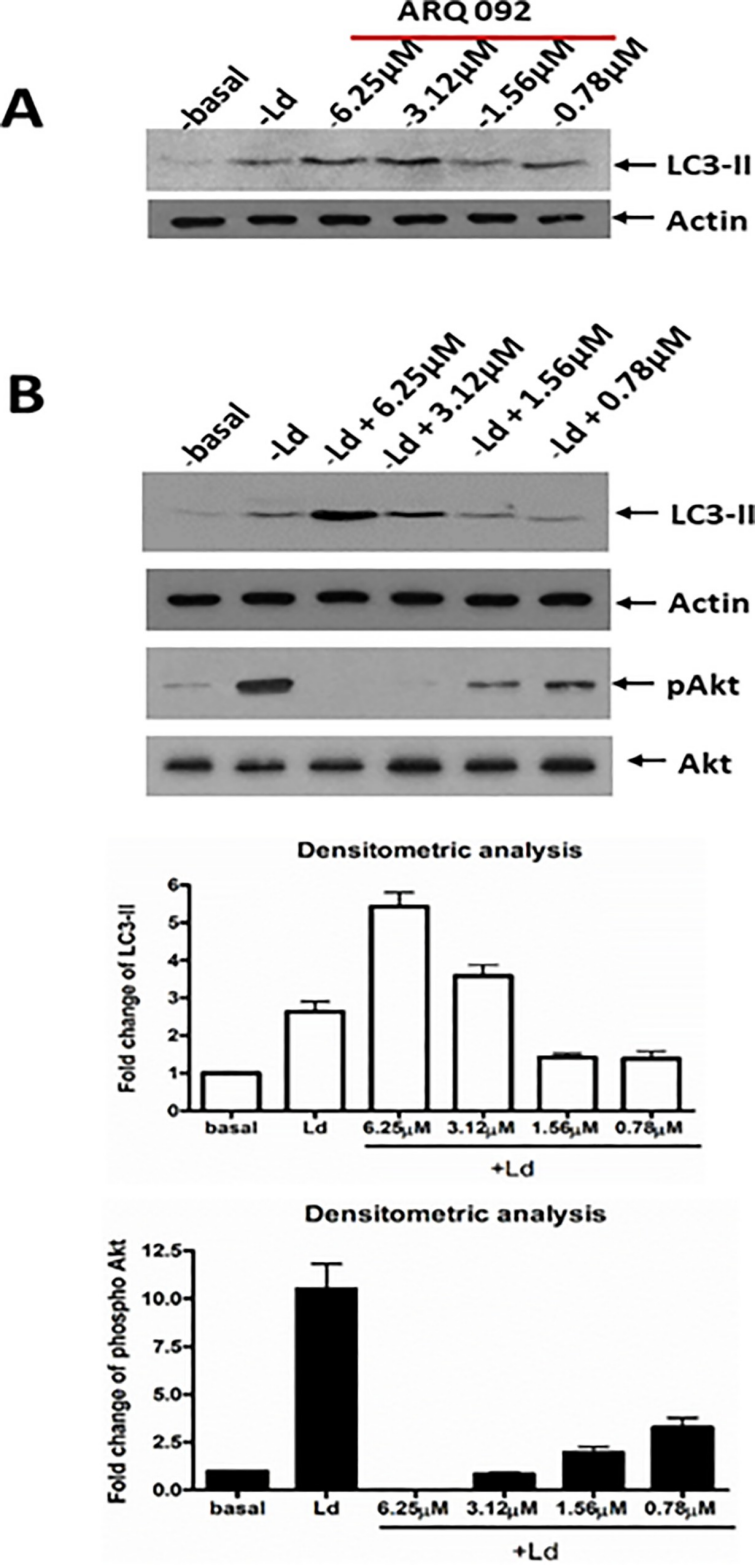
Leishmania induced autophagy is enhanced in the presence of Miransertib by inhibiting pAkt. dTHP-1 cells were incubated with *L*. *donovani* promastigotes for 24 h. After 24 h infection, cells were treated with indicated concentrations of Miransertib for an additional 24 h (panel **B**). In parallel, non-infected cells were treated with Miransertib alone for the comparison (panel **A**), and infected cells without Miransertib were used as controls. At the end of the experiment, cells were then washed with HBSS and whole cell lysates were collected and analyzed by immunoblotting for LC3-II, pAkt and Akt (levels (panel **B**). Actin levels were also analyzed as loading controls. The histograms shown are densitometric analyses of relative LC3-II levels and pAkt in three independent experiments. Data are presented as mean ± SD. Densitometric analysis was performed in ImageJ. The results of LC3-II are expressed normalized to actin where as the results of pAkt are expressed normalized to Akt.

To evaluate further anti-leishmanial mechanism(s) of Miransertib, we looked for macrophage biological pathways possibly influenced by *Leishmania*-mediated activation of Akt. One intriguing target is the autophagic pathway which can be inhibited by the activation of Akt through mammalian target of rapamycin (mTOR) [25]. Notably, autophagy has been shown to play a role in the pathogenesis of *Leishmania* infection [18,26,27]. In order to investigate whether post treatment of *L*. *donovani* infected cells with Miransertib affects host autophagy, cells infected for 24 hours were treated with indicated concentrations of Miransertib for an additional 24 hours and analyzed for LC3-II levels. The results presented in Fig 5 show that each alone—infection or inhibitor treatment—were equipotent, and together they were additive in boosting LC3-II levels.

In order to confirm that Miransertib was effective in attenuating phosphorylation of macrophage Akt during infection, we also analyzed whole cell lysates from non-infected cells, *Leishmania* infected cells, and infected and Miransertib-treated cells for the level of phosphorylated Akt. As expected, *Leishmania* markedly enhanced the level of macrophage phosphorylated Akt (Fig 5, panel B) and Miransertib treatment attenuated the phosphorylation of macrophage Akt in infected cells in a concentration dependent manner (Fig 5, panel B).

**Fig 6:**
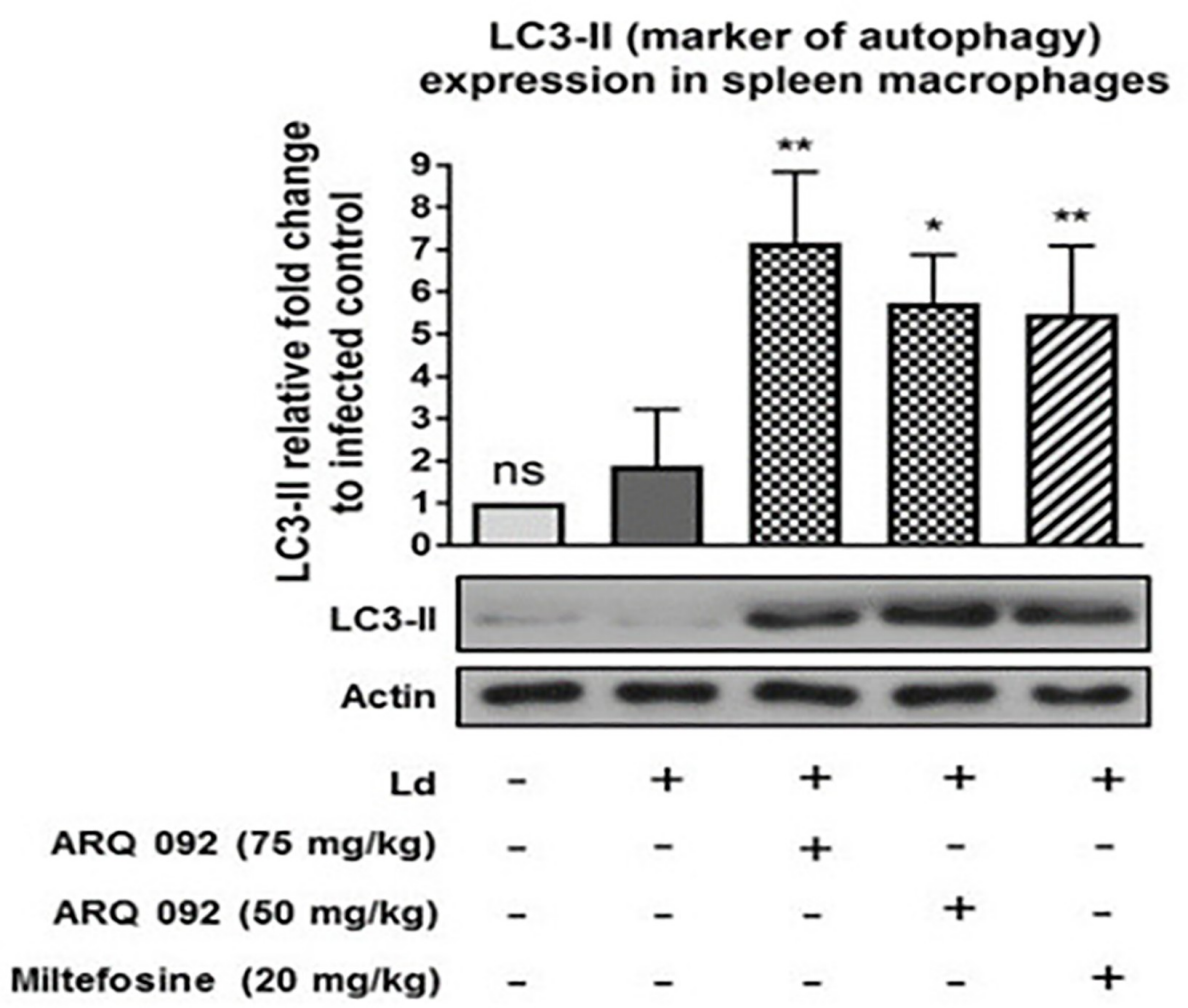
Miransertib treatment enhances the level of macrophage autophagy in *Leishmania* infected mice spleens. Macrophages from *Leishmania*-infected mice spleens, and *Leishmania*-infected and treated with Miransertib or miltefosine mice spleens were isolated as described in “Materials and Methods” and analysed for the levels of LC3-II. The histogram shows the levels of LC3-II in macrophages of Miransertib-treated or Miltefosine-treated leishmania infected spleens normalized to LC3-II levels in non-treated leishmania infected spleens. Data are presented as mean ± SD of macrophages obtained from spleens of 4 mice in each group. Densitometric analysis was performed in ImageJ. The results of LC3-II are expressed normalized to actin.

To support the above *in vitro* finding that Miransertib treatment was additive to macrophage autophagy induced in response to *L*. *donovani* infection, we investigated whether Miransertib administration promoted autophagy *in vivo* in *Leishmania* infected organs. After infection, one of the organs with high parasite burdens is the spleen. We isolated macrophages from spleens of mice infected with *L*. *donovani* and treated or not with Miransertib to examine levels of LC3-II. As shown in Fig 6, macrophages isolated from the spleens of infected and Miransertib treated mice showed a significantly higher level of LC3-II than the cells from infected spleens without Miransertib treatment. Taken together, these results suggest that potentiation of autophagy in infected cells by Miransertib could be one mechanism responsible for its leishmaniacidal activity.

## Discussion

Drugs available for the treatment of leishmaniasis have numerous drawbacks including limited efficacy, significant toxicities, development of parasite resistance and generally poor oral bioavailability, amongst others. In the present study, we examined the anti-leishmanial efficacy of Miransertib, a potent, orally available Akt inhibitor [17]. Here we show that Miransertib inhibited the growth of *Leishmania* promastigotes in culture and amastigotes in macrophages and reduced parasite burdens and the size of lesions in experimental visceral and cutaneous models of leishmaniasis.

The rationale to examine an Akt inhibitor as a potential candidate to treat leishmaniasis came from our recent studies that *Leishmania* activates Akt signaling in infected cells [5,7,8] to promote the persistence of the infection. A few reports have shown that Akt is activated by *Leishmania* infection and that knock down of Akt limits survival of *Leishmania* parasites in infected cells [6,11,13,18] also provided evidence that Akt is the most likely target of miltefosine in *Leishmania* infections. Together, those studies demonstrated that Akt plays an essential role in *Leishmania* pathogenesis and that its inhibition is a viable approach to control *Leishmania* infections. We elected to study Miransertib for multiple reasons. Miransertib is a selective Akt inhibitor currently in phase Ib clinical trials for the treatment of lymphoma, breast and endometrial cancers, and tumors with Akt or phosphoinositide 3-kinase (PI3K) mutations. In fact, several Akt inhibitors, including Miransertib, are being developed as anti-cancer drugs [14,15,17]. Furthermore, Miransertib is orally bioavailable, stable in acidic environments (stable at pH 2.2 for *at least* 28 days) and heat stable (40°C/75% relative humidity for *at least* 6 months) (unpublished data). Thus, its chemical stability is compatible with its use in endemic areas. For all these reasons, Miransertib was selected as an interesting drug candidate for leishmaniasis. It was our hypothesis that inhibiting host Akt signaling with this drug would be detrimental to *Leishmania* survival, and this turned out to be correct. It should be recognized that in contrast to malignant cells and cells infected by *Leishmania* or other pathogens that induce sustained Akt activation, non malignant cells will not be affected by Akt inhibition because in most cells, Akt is predominantly in the inactive state. Re-purposing of existing anti-Akt drug candidates in clinical trials can be an attractive option as *de novo* drug discovery programs are prohibitively expensive and time consuming.

The present study shows that Miransertib has potent and direct inhibitory effects on *Leishmania* promastigote forms and kills the intracellular forms of *L*. *donovani* and *L*. *amazonensis* at low micro molar concentrations. Regarding the mechanisms of leishmaniacidal activity of Miransertib on *in vitro* promastigotes forms, it is likely that this Akt inhibitor reduces the activity of an Akt ortholog of *L*. *donovani* and *L*. *amazonensis*, which is essential for the parasite survival. No high identity/homology Akt protein has been identified in *Leishmania* thus far. However, while this manuscript was in preparation, it was reported that *Leishmania spp*. express a novel gene, named Ld-RAC/Akt-like gene, from *L*. *donovani* [28] and Lp-RAC/Akt-like gene from *L*. *panamensis* [29]. Although this gene exhibits 26.5% identity with mammalian Akt1, its protein product contains major mammalian Akt hallmarks, including the typical pleckstrin homology domain, protein kinase and AGC kinase domains [29]. According to the comparative analysis in Clustal W the Akt-like protein of *L*. *panamensis* is highly conserved amongst the different species of *Leishmania*, indicating that this protein may be essential for *Leishmania* survival. It is of interest, that this protein has been suggested as a druggable target in anti-*Leishmania* therapy. Taken together, it is tempting to speculate that in addition to its inhibitory activity on host Akt, Miransertib may also exert a direct inhibitory effect on the parasite’s Akt-like activity. Together, Miransertib leads to suppression of *Leishmania* survival and growth.

The *in vitro* results reported above strongly support the candidacy of Miransertib as a potential anti-*Leishmania* therapeutic. Nevertheless, it was necessary to investigate the efficacy of Miransertib against amastigotes inside infected host cells. Our results presented in Fig 2A and 2B clearly show that Miransertib is highly active against intracellular *L*. *donovani* and *L*. *amazonensis* amastigotes at concentrations well below cytotoxic levels and effective concentration against the promastigotes. As for the mechanism(s) of leishmaniacidal activity of Miransertib on intracellular amastigotes, it is possible that cell permeable Miransertib can directly affect intracellular amastigote survival by inhibiting the *Leishmania* Akt ortholog. However, it is also possible that Miransertib blocks macrophage Akt associated cell signaling pathways that are required to create a niche for *Leishmania* survival. This hypothesis is based on our recent finding that *Leishmania* activates Akt signaling in macrophages, which promotes the survival of intracellular *Leishmania* [5,30]. Akt is a key molecule involved in a large number of important biological processes [9,10]. Amongst these, autophagy [18,26,27] is particularly relevant to *Leishmani*a pathogenesis. Akt is known to negatively regulate autophagy by promoting activity of mTOR [25]. Thus, it was of interest to investigate whether Miransertib promotes autophagy as this could contribute to its anti-*Leishmania* activity. This seemed to have particular relevance since host autophagy is involved in host defense against intracellular pathogens [31] including *Leishmania* [18,27]. The results in Fig 5 indeed show that Miransertib induces autophagy, likely via mTOR, in infected cells, and this may represent one mechanism of Miransertib mediated killing of intracellular *Leishmania*.

Next, we examined the efficacy of Miransertib *in vivo* in experimental models of visceral and cutaneous leishmaniasis. In the visceral leishmaniasis model, the impact of Miransertib was evaluated and compared with the reference drug miltefosine (Fig 3A and 3B). Miransertib at 50 and 75 mg/kg doses brought about > 80% reduction in parasite burdens in the spleen and liver where it appeared to outperform miltefosine (Fig 3B), further supporting the therapeutic potential of Miransertib. Regarding potential mechanism(s) of action of Miransertib *in vivo*, we analyzed the level of autophagy in the spleens of *L*. *donovani* infected and Miransertib treated mice. Similar to the results obtained using *Leishmania*-infected macrophages *in vitro* (Fig 5), the results presented in Fig 6 illustrate that Miransertib elevated autophagy, likely via mTOR, in infected spleens and may represent one mechanism of action of Miransertib mediated killing of *Leishmania in vivo*. The support for this possibility comes from our recent finding that *L*. *donovani* actively inhibits mTOR dependent autophagy by sustained activation of macrophage Akt [18]. Taken together, the data show that Miransertib is effective in the treatment of *L*. *donovani* infected BALB/c mice by oral administration, without noticeable toxicity. In the cutaneous model of leishmaniasis, Miransertib treatment led to up to 40% reduction of lesion size. Miltefosine was more effective in this experimental model suggesting that it may accumulate at cutaneous sites with greater efficiency than that of Miransertib, which was tested at higher concentration as compared to the maximum concentration used in the visceral model. The difference between the efficacy of Miransertib and miltefosine in this experimental model of cutaneous leishmaniasis suggests that there are differences in the distribution of these compounds to cutaneous tissues. Our studies provide important evidence to support further study of this Akt-specific inhibitor as a novel therapeutic for treatment of leishmaniasis.

Recently, an alkylphospholipid analog edelfosine (1-*O*-octadecyl-2-*O*-methyl-*rac*-glycero-3-phosphocholine) has been shown be effective against *Leishmania* spp [32]. Previously, edelfosine has been shown to inhibit PI3K/Akt survival pathway by displacing Akt as well as key regulatory kinases p-PDK1 (phosphatidylinositol-dependent protein kinase 1), PI3K and mTOR from lipid rafts in mantle cell lymphoma cells [33]. Regarding its mode of action, edelfosine has been shown to accumulate in cholesterol- and sphingolipid-rich rafts, in part, due to its high affinity for cholesterol and possibly results in displacement of Akt and the kinases PI3K, PDK1 and mTOR from lipid rafts. In contrast, Miransertib is a highly specific Akt inhibitor.

In summary, the data reported here show that Miransertib possesses potent leishmanicidal activity against *L*. *donovani* and *L*. *amazonensis* in both promastigote and intracellular amastigote forms *in vitro* and *in vivo* at concentrations that are not toxic to the host. The anti-leishmanial activity of Miransertib appears to be associated with the targeting of Akt and the induction of host autophagy, which may represent one mechanism for resolution of this disease. Our findings strongly support that Miransertib should be considered for further evaluation in clinical trials of leishmaniasis.

## Supporting information

S1 Fig Miransertib (ARQ 092) pretreatment attenuates LPS induced phosphorylation of macrophage Akt. dTHP-1 cells were treated with indicated concentrations of Miransertib for 4 h followed by stimulation with LPS (100 nM) for 45 min. Whole cell lysates from non-treated and Miransertib treated cells were analyzed with the indicated antibodies. Shown is a representative western blot for pAkt and Akt levels of two independent experiments.
(TIF)

S2 Fig Miransertib treatment induces macrophage autophagy. dTHP-1 cells were treated with indicated concentrations of Miransertib for 24 h. Whole cell lysates from non-treated and Miransertib treated cells were collected and analyzed by immunoblotting for LC3-II as a marker of autophagy. The same membrane was stripped and reprobed for actin as a loading control. Shown is a representative western blot for LC3-II and actin levels of two independent experiments.
(TIF)

S3 Fig Evaluation of human macrophage toxicity of Miransertib. dTHP-1 cells were incubated with indicated concentrations of Miransertib for 24 h. For toxicity assessment, cell proliferation activity was evaluated using MTS reagent as described in “Materials and Methods”. The histogram shows the percent change in OD at 490 mm in Miransertib treated cells normalized to non-treated ones in three independent experiments performed in duplicate. Data are presented as mean ± SD.
(TIF)
