# Supplementary figures and images for: Miransertib (ARQ 092), an orally-available, selective Akt inhibitor is effective against Leishmania
Source: PLoS One. 2018 Nov 6;13(11):e0206920. doi: 10.1371/journal.pone.0206920 (PMC6219794; doi:10.1371/journal.pone.0206920)

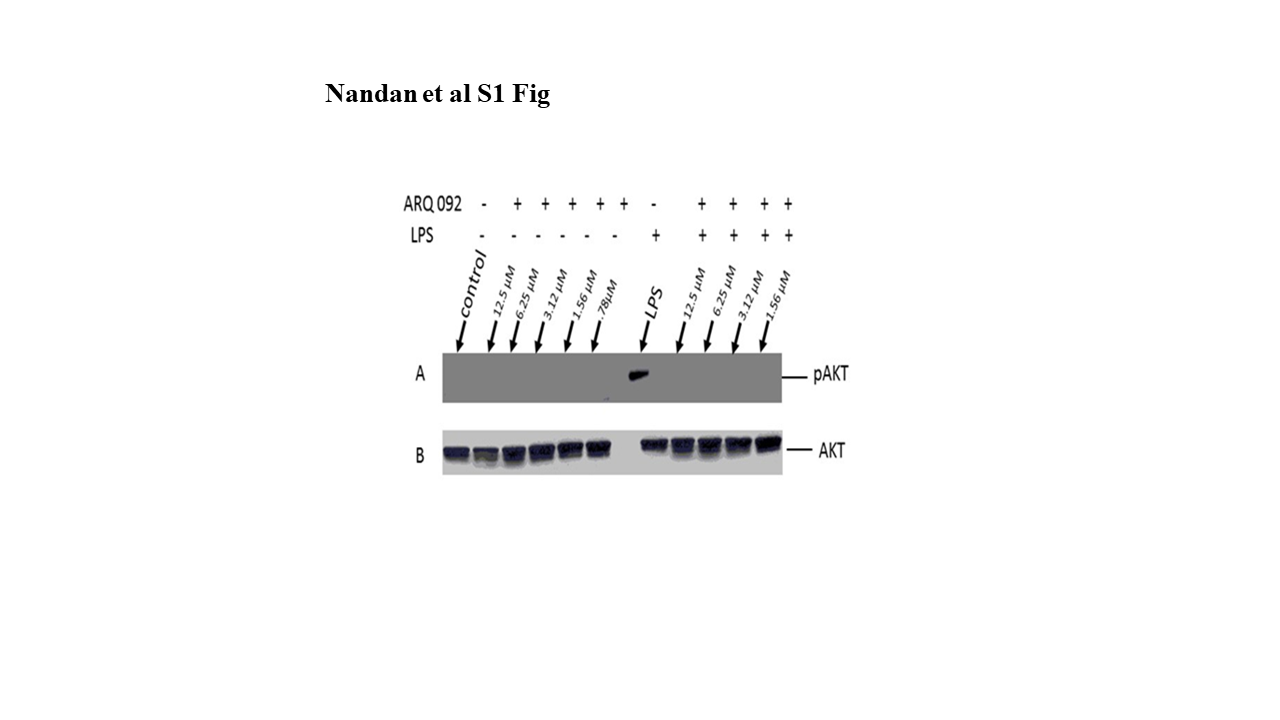

Supplement: S1 Fig — dTHP-1 cells were treated with indicated concentrations of Miransertib for 4 h followed by stimulation with LPS (100 nM) for 45 min. Whole cell lysates from non-treated and Miransertib treated cells were analyzed with the indicated antibodies. Shown is a representative western blot for pAkt and Akt levels of two independent experiments. (TIF) [file pone.0206920.s001.tif]

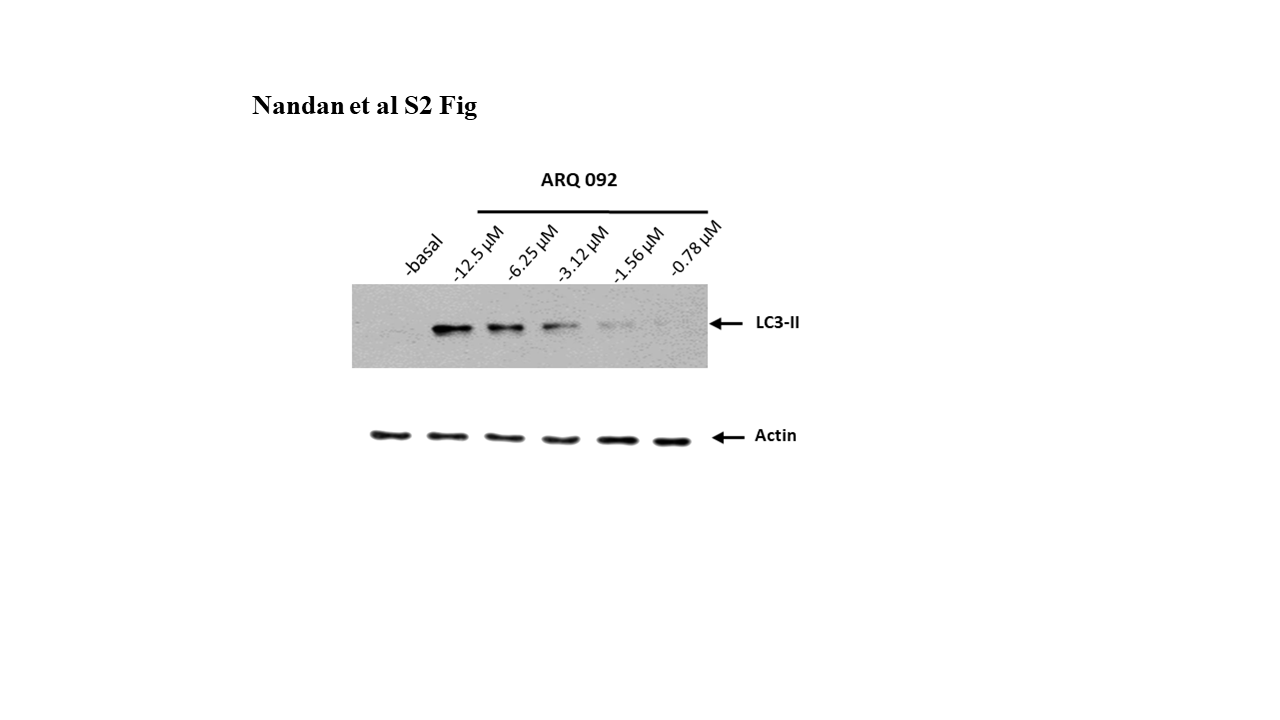

Supplement: S2 Fig — dTHP-1 cells were treated with indicated concentrations of Miransertib for 24 h. Whole cell lysates from non-treated and Miransertib treated cells were collected and analyzed by immunoblotting for LC3-II as a marker of autophagy. The same membrane was stripped and reprobed for actin as a loading control. Shown is a representative western blot for LC3-II and actin levels of two independent experiments. (TIF) [file pone.0206920.s002.tif]

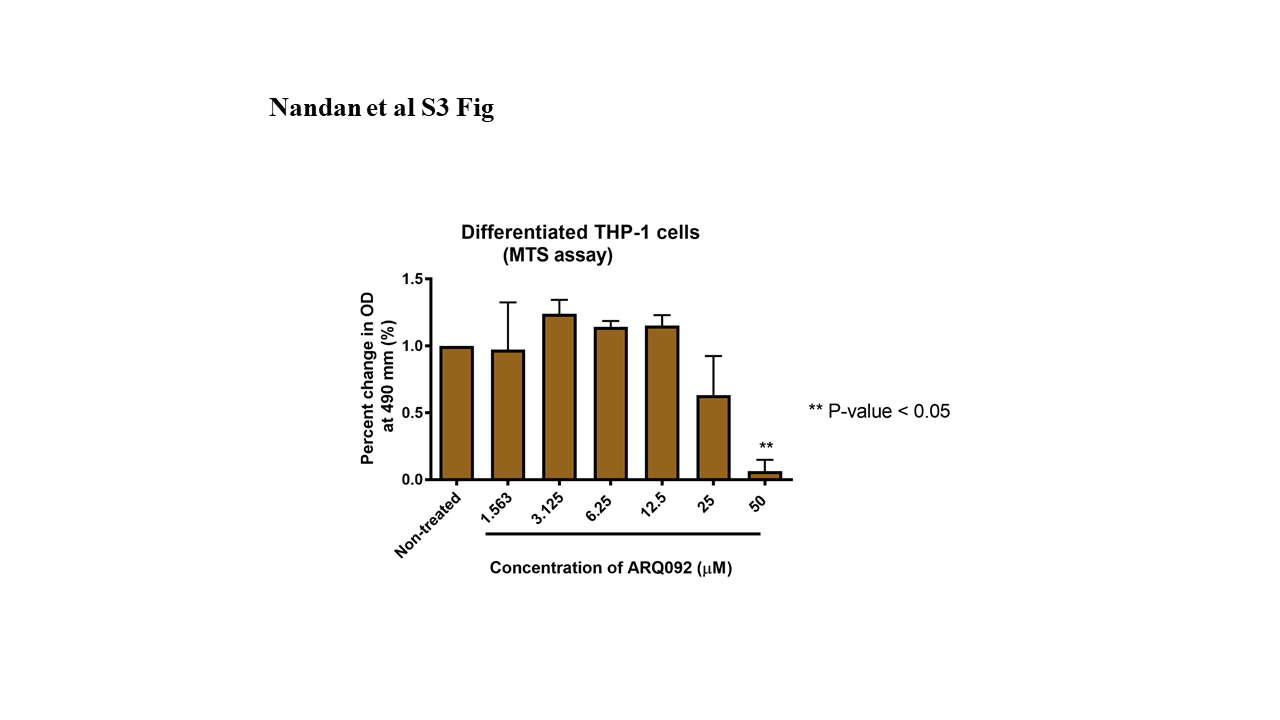

Supplement: S3 Fig — dTHP-1 cells were incubated with indicated concentrations of Miransertib for 24 h. For toxicity assessment, cell proliferation activity was evaluated using MTS reagent as described in “Materials and Methods”. The histogram shows the percent change in OD at 490 mm in Miransertib treated cells normalized to non-treated ones in three independent experiments performed in duplicate. Data are presented as mean ± SD. (TIF) [file pone.0206920.s003.tif]
